# Fecal microbiota and bile acid interactions with systemic and adipose tissue metabolism in diet-induced weight loss of obese postmenopausal women

**DOI:** 10.1186/s12967-018-1619-z

**Published:** 2018-09-03

**Authors:** José O. Alemán, Nicholas A. Bokulich, Jonathan R. Swann, Jeanne M. Walker, Joel Correa De Rosa, Thomas Battaglia, Adele Costabile, Alexandros Pechlivanis, Yupu Liang, Jan L. Breslow, Martin J. Blaser, Peter R. Holt

**Affiliations:** 1 Rockefeller University, 1230 York Avenue, New York, NY 10065 USA; 2 New York University Langone Medical Center, 423 East 23rd St, New York, NY 10016 USA; 3 The Pathogen and Microbiome Institute, Northern Arizona University, Flagstaff, AZ USA; 4 Division of Computational and Systems Medicine, Department of Surgery and Cancer, Imperial College London, London, S1W7 2AZ UK; 5 Department of Food and Nutritional Sciences, School of Chemistry, Food and Pharmacy, University of Reading, Reading, RG6 6AP UK

**Keywords:** Obesity, Diet-induced weight loss, Plasma metabolome, Fecal microbiota, Fecal bile acids, Correlation analysis, Gut microbiota-fecal bile acids, Gut microbiota-plasma metabolome, Fecal bile acids-plasma metabolome, Fecal microbiota-adipose tissue transcriptome

## Abstract

**Background:**

Microbiota and bile acids in the gastrointestinal tract profoundly alter systemic metabolic processes. In obese subjects, gradual weight loss ameliorates adipose tissue inflammation and related systemic changes. We assessed how rapid weight loss due to a very low calorie diet (VLCD) affects the fecal microbiome and fecal bile acid composition, and their interactions with the plasma metabolome and subcutaneous adipose tissue inflammation in obesity.

**Methods:**

We performed a prospective cohort study of VLCD-induced weight loss of 10% in ten grades 2–3 obese postmenopausal women in a metabolic unit. Baseline and post weight loss evaluation included fasting plasma analyzed by mass spectrometry, adipose tissue transcription by RNA sequencing, stool 16S rRNA sequencing for fecal microbiota, fecal bile acids by mass spectrometry, and urinary metabolic phenotyping by ^1^H-NMR spectroscopy. Outcome measures included mixed model correlations between changes in fecal microbiota and bile acid composition with changes in plasma metabolite and adipose tissue gene expression pathways.

**Results:**

Alterations in the urinary metabolic phenotype following VLCD-induced weight loss were consistent with starvation ketosis, protein sparing, and disruptions to the functional status of the gut microbiota. We show that the core microbiome was preserved during VLCD-induced weight loss, but with changes in several groups of bacterial taxa with functional implications. UniFrac analysis showed overall parallel shifts in community structure, corresponding to reduced abundance of the genus *Roseburia* and increased *Christensenellaceae;g__* (unknown genus). Imputed microbial functions showed changes in fat and carbohydrate metabolism. A significant fall in fecal total bile acid concentration and reduced deconjugation and 7-α-dihydroxylation were accompanied by significant changes in several bacterial taxa. Individual bile acids in feces correlated with amino acid, purine, and lipid metabolic pathways in plasma. Furthermore, several fecal bile acids and bacterial species correlated with altered gene expression pathways in adipose tissue.

**Conclusions:**

VLCD dietary intervention in obese women changed the composition of several fecal microbial populations while preserving the core fecal microbiome. Changes in individual microbial taxa and their functions correlated with variations in the plasma metabolome, fecal bile acid composition, and adipose tissue transcriptome.

*Trial Registration* ClinicalTrials.gov NCT01699906, 4-Oct-2012, Retrospectively registered. URL-https://clinicaltrials.gov/ct2/show/NCT01699906

**Electronic supplementary material:**

The online version of this article (10.1186/s12967-018-1619-z) contains supplementary material, which is available to authorized users.

## Background

The fecal microbiome has emerged as a potential area of research to address the obesity epidemic in the developed world [1–4]. Dietary manipulations for weight reduction that emphasize differing macro nutrient composition generally have failed to cause sustained weight loss, instead leading to large but temporary changes in microbiota composition [5]. Caloric restriction by medical or surgical measures is successful in lowering weight and modulating or eliminating several obesity complications such as type 2 diabetes, hepatic steatosis and cardiovascular disease [6, 7]. Rapid weight loss using a very low calorie diet (VLCD) is effective in short term studies or to prepare morbidly obese subjects for bariatric surgery [8]. These diets typically are very low in carbohydrate and fat content while keeping protein content optimal.

We recently completed a study providing a VLCD fed to ten class 2–3 obese post-menopausal women (BMI > 35 kg/m^2^) in the metabolic facilities of the Rockefeller University Hospital to achieve rapid weight loss of 10% of baseline weight in a mean of 46 days [9]. This intervention reduced circulating leukocytes, high-sensitivity C-reactive protein (hsCRP), as well as IL6 and improved insulin sensitivity. A surprising finding was an increase in subcutaneous adipose tissue crown-like structures (CLS) not accompanied by change in inflammatory gene expression, but associated with increased circulating fatty acids, glycerol, and ketone bodies indicative of rapid lipolysis. We suggested that this was part of an adipose tissue remodeling process. Numerous fasting plasma metabolites were significantly altered after weight loss. As part of that study, we collected fresh fecal samples for analyses of microbiota and bile acids at baseline and after the planned weight loss had been achieved.

Obese individuals have been reported to have different microbial richness and composition than normal weight individuals [10], and their gut microbiota may harvest more calories from the diet [11]. The metabolic changes of obesity may be accompanied by altered gastrointestinal microbiota [12–14] and fecal excretion of differing bile acids [15]. Furthermore, weight loss by medical [16] or surgical means [17] profoundly affects bile acid homeostasis as well as fecal microbiota and short chain fatty acid composition. Gut microbiota appear to have a major effect on circulating metabolites [18] and thus can affect caloric utilization through gut-brain interactions [13, 14]. The concentration and composition of bile acids in the gastrointestinal track also affect carbohydrate and lipid metabolism and may play a role in altering rates of weight loss [19].

The microbiota and bile acids present in the gut lumen have profound roles in determining systemic metabolism [20, 21]. Although changes in fecal microbiota have been correlated with plasma metabolites in obesity and weight loss [18, 22], the interactions of microbiota and bile acids in the gut lumen with circulating metabolites and adipose tissue metabolism in humans are unknown. In the current study, we examined the effects of VLCD-induced 10% weight loss in obese women upon fecal microbiota and bile acid content and composition. We hypothesized that VLCD-induced weight loss would have a modest but measureable effect on fecal microbiota with associated changes in fecal bile acids in response to nutrient restriction. We further investigated the interrelationship between changes in fecal microbiota and fecal bile acids, as well as the effects on circulating metabolites and subcutaneous adipose tissue. Following VLCD-induced weight loss, we found linked changes in the fecal microbiome, plasma metabolome, and adipose tissue transcriptome.

## Methods

The details of this clinical study have been published [9]. In brief, eligible subjects were obese (with a BMI ≥ 35 at the time of screening) postmenopausal women. Subjects who had clinical cardiovascular disease (CVD); had type 2 diabetes; were on oral hypoglycemic agents; were smokers; were regular users of aspirin, fish oils or vitamin D supplements; had a history of gastrointestinal surgery other than appendectomy; or had cancer were excluded. Subjects with antibiotic use within 3 months or during the study were excluded. Of 12 subjects enrolled, 10 completed the study. In this single center study performed at The Rockefeller University Hospital, subjects underwent a complete medical examination, standard blood and urine tests, and an electrocardiogram; all were found to be healthy. The sample size for the original study [9] was derived to determine whether loss of 10% of baseline weight would significantly change the density of CLS in adipose tissue, but was also influenced by logistical and feasibility considerations.

### Trial approval and registration

The study was approved by the Institutional Review Boards at Rockefeller University, Weill Cornell Medical College, and Memorial Sloan Kettering Cancer Center, and registered under ClinicalTrials.gov identifier NCT01699906.

### Interventions

Participants were admitted to the Rockefeller University Hospital for baseline testing and underwent an initial 3-day dietary adjustment period during which they consumed 50% of their calculated caloric intake based on their pre-study diet assessment followed by the 800 kcal/day VLCD. After losing approximately 10% of their baseline body weight, subjects continued to consume the VLCD for 3 days, when all of the baseline measurements were repeated.

The VLCD consisted of a commercially available diet (New Direction Program Robard Corporation, Mount Laurel NJ) that provided approximately 800 kcal/day with an estimated macronutrient energy distribution of 54% protein, 26% carbohydrate, 20% fat [23]. This commercial preparation provided a choice of shakes, soups, bars, and puddings. Subjects had 4 meal choices per day and consumed one item every 4 waking hours.

### Baseline and final testing procedures

Fasting blood samples were analyzed in the Clinical Pathology Laboratory of the Memorial Sloan Kettering Cancer Center for electrolytes, liver function, renal function, lipid profile, and hsCRP. Body composition was measured using the BodPod Tracking system based on air displacement plethysmography (COSMED, Italy). Subcutaneous aspiration adipose tissue biopsies were performed and were frozen in RNA later for subsequent RNA extraction and analysis. A baseline stool sample was collected within the first 2 days after admission and a final sample taken during the last 3 days of the final evaluation. Immediately after collection, the stool samples were snap-frozen and kept at − 80 °C until analysis. Plasma FGF19 levels were measured by ELISA (R&D systems, Minneapolis MN) following the manufacturer’s instructions.

### Statistical analyses

Two-tailed paired t-tests and Wilcoxon tests were used to compare anthropometric measurements, biochemical variables, serum cytokines, and immunohistochemical values before and after VLCD-induced weight loss, with p < 0.05 considered significant. GraphPad Prism and Excel were used for data visualization. Statistical analyses for the individual methods are described below.

### Fecal microbiome analyses

Fecal samples were collected and snap-frozen and DNA prepared using a phenol–chloroform extraction technique with bead beating for mechanical disruption. Purified DNA was PCR-amplified using modified universal primers for the V4–V5 portion of the 16S ribosomal RNA gene, per Additional file 1: Table S1. The purified PCR products were pooled and barcoded following the Illumina TruSeq Sample Preparation protocol and examined using the Illumina MiSeq sequencing platform.

Microbiome sequencing data were analyzed with QIIME2 2017.2 (https://qiime2.org), a plugin-based microbiome analysis platform [24]. Raw sequence data were demultiplexed and quality filtered using the q2-demux plugin. This was followed by denoising with DADA2 [25] with the q2-dada2 plugin for quality filtering and identification of sequence variants (i.e., 100% operational taxonomic units). Sequence variants were aligned with mafft [26] (using the q2-alignment plugin) and used to construct a phylogeny with fasttree2 [27] (using the q2-phylogeny plugin). Alpha-(observed OTUs, Faith’s Phylogenetic Diversity [28]) and beta-diversity metrics (weighted and unweighted UniFrac [29]) and principal coordinate analysis (PCoA) were calculated using the q2-diversity plugin at a depth of 8000 sequences per sample. Paired difference testing between pre- and post-VLCD groups was performed with the q2-longitudinal plugin [30]. Sequence variants were taxonomically classified using classify-sklearn [31] against the Greengenes 13_8 99% OTUs reference sequences [32]. All downstream analyses of taxonomic composition were performed at genus level. We used paired Wilcoxon signed rank tests to determine whether the relative abundances of individual microbial taxa differed between paired baseline and post-VLCD time points within individual subjects. PERMANOVA tests with 999 permutations were used to determine whether between-sample distances were significantly different between pre- and post-VLCD groups and between subjects [33]. Total bacterial count was measured by PCR of bacterial 16S rRNA gene sequences as previously described [34].

### Metabolomic analyses

#### Plasma metabolomics by mass spectrometry

Plasma samples were analyzed using a commercially available platform (Metabolon, Durham NC) for detection of biochemicals of known identity using liquid chromatography–mass spectrometry (LC–MS) and gas chromatography–mass spectrometry (GC–MS). Matched paired t-tests were used to identify compounds that differed significantly between the pre- and post-weight loss samples correcting for multiple measurements.

#### Urinary metabolomics by ^1^H NMR spectroscopy

Urine metabolic phenotypes were measured by ^1^H NMR spectroscopy using a 700 MHz Bruker NMR spectrometer, operating at 300 K and equipped with a 5 mm ^1^H(^13^C/^15^N) inverse cryoprobe. All samples were prepared using methods as described [35]. For each urine sample, a standard one-dimensional NMR spectrum was acquired with water peak suppression using a standard pulse sequence. For each spectrum, 8 dummy transients and 128 transients were collected into 64 K data points with a spectral width of 12.001 ppm, and all spectra were manually corrected for phase and baseline distortions. Urine samples were referenced to the TSP singlet at δ 0.0. An in-house Matlab (version R2009b, The Mathworks, Inc.; Natwick MA) script was used to digitize the spectra. The region containing the water resonance was removed from the spectra to minimize the baseline distortions resulting from imperfect water saturation.

Intervention-associated metabolic variation was investigated in the urinary metabolic profile using orthogonal projection to latent structures-discriminant analysis (OPLS-DA) constructed using unit variance scaling using in-house Matlab scripts. ^1^H NMR spectroscopic profiles were used as the descriptor matrix (X) and class membership (sampling point: pre = 0, post-intervention = 1) as the dummy matrix (Y). Loading coefficient plots were generated by back-scaling transformation to display the covariance between the Y-response matrix (pre/post-intervention) and the signal intensity of the metabolites in the NMR data (X). Hence, the direction and magnitude of the signals relate to the covariation of the metabolites with the Y-response in the model. Colors projected onto the coefficient plot indicate the correlation coefficient (r^2^) between each metabolite and the Y-response variable, with red indicating strong significance and blue indicating weak significance. The predictive performance (Q^2^Y) of the model was calculated using a sevenfold cross validation approach and model validity was established by permutation testing (10,000 permutations), with results given as *p* values.

#### Fecal bile acid measurements by ultra performance liquid chromatography–mass spectrometry (UPLC–MS)

Fecal samples were prepared by combining 100 mg of dried feces with 2 mL of NaOH (0.1 mol/L) and then incubating at 60 °C for 1 h. Water (4 mL) was then added to the samples followed by homogenization using a Polytron homogenizer (Kinematica GMBH, Lucerne, Switzerland) set at maximal speed for 30 s. Samples were spun at 20,000×*g* for 20 min and the supernatant was removed. Bile acids were measured in the supernatant as described [36], using ACQUITY ultra performance liquid-chromatography (UPLC) (Waters Ltd., Elstree, UK) coupled to a Xevo G2 Q-ToF mass spectrometer (Waters, Manchester, UK) equipped with an electrospray ionization source operating in negative mode. TargetLynx application manager, a module of the MassLynx software, was used for the processing and quantification of the results. In total, 25 bile acids were quantified in the fecal samples using the method created from the retention times and mass spectra acquired from authentic standard solutions, analyzed in the same conditions as the samples. Results are presented as mass spectrometry counts or ratio from post weight loss (PostWL)/preweight loss (PreWL) samples.

### Pairwise correlation analyses statistics

**Fig. 1:**
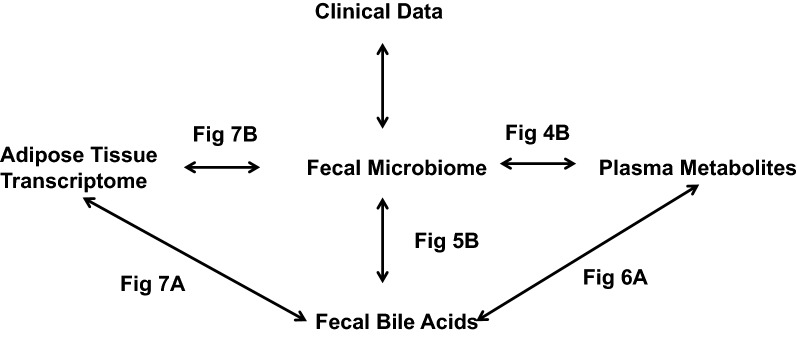
Outline of pairwise correlation analyses performed

Plasma metabolites that were significantly changed from pre- to post-weight loss (paired *t* test corrected for multiple comparisons p < 0.05) were included. Bacterial taxa were filtered by significant change from pre- to post-weight loss with a magnitude of change > 30% from pre- to post-weight loss. Fecal bile acids that were significantly changed from the pre- to post-weight loss were included in this analysis (paired t-test with Tukey’s correction p < 0.05). Pairwise correlation analyses between plasma metabolites and fecal bacterial taxa, fecal bile acids and fecal bacterial taxa, plasma metabolites and fecal bile acids, adipose tissue transcriptome and fecal bile acids, adipose tissue transcriptome and fecal bacterial taxa were carried out by cross-product correlation and displayed as a heatmap using R. We outline the analyses presented in this study in Fig. 1. A correlogram was constructed for each binary dataset comparison in R to display the correlation matrix calculated using Spearman correlation p-value < 0.1 corrected for multiple testing. For every pairwise comparison (e.g. bile acids and bacterial taxa), positive correlations (p < 0.1) are indicated by red and negative correlations by blue, while strongly positive associations (p < 0.05) are denoted with dark red and strongly negative associations are denoted with dark blue. Bubble plots were used to display the magnitude of the correlation (r-value) against the significance of such correlation (p-value) for a given feature or feature set.

## Results

The detailed description of the clinical, plasma metabolome, and adipose tissue transcriptome results of this study have been reported [9]. Summarizing these prior results, the VLCD, low in carbohydrate and fat content but with adequate protein content, induced ~ 10% weight loss in a mean of 46.2 ± 15.3 days. Most (85%) of the weight loss was fat, with the remainder of weight lost being water and lean protein. Weight loss was accompanied by improved insulin sensitivity, a pronounced fall in serum leptin, and decreased WBC and hsCRP, but circulating uric acid levels did not change significantly (PreWL 6.2 ± 0.2, PostWL 5.9 ± 0.4 p = 0.29). In adipose tissue, CLS density increased without concomitant changes in adipocyte diameter or in inflammatory gene expression pathways. However, the expression of gene pathways involved in fatty acid and phospholipid metabolism increased with parallel decreases in fatty acid synthesis gene expression. In addition, of 336 plasma metabolites detected, 131 changed significantly, with 67 increasing and 64 decreasing during weight loss. This included increased levels of glycerol and ketones such as β-hydroxybutyrate (BHB) (16-fold increase), consistent with starvation ketosis.

Newly reported in this manuscript, several metabolic changes were detected by urinary metabolic phenotypes before and after VLCD-induced weight loss using ^1^H NMR spectroscopy (Additional file 1: Fig. S1). The partial least squares-discriminant analysis (PLS-DA) model comparing the metabolic profiles between the sampling points had strong predictive power (Q^2^Y = 0.56; p ≤ 001), indicating clear metabolic differences following weight loss. Consistent with the plasma data, urine concentrations of acetoacetate and acetone were increased post-VLCD, consistent with starvation ketosis. 3-Methyl histidine and creatine, metabolites related to muscle protein catabolism, decreased following the VLCD, suggestive of protein-sparing. Alanine and taurine also showed diminished excretion. The amino acid isoleucine and its breakdown products 3-methyl-2-oxovalerate 3-hydroxyisovalerate increased in urine after VLCD-induced weight loss. Microbial derived co-metabolites of tyrosine and phenylalanine, 4-cresyl sulfate (4CS) and phenylacetylglutamine (PAG), increased in the urine whereas the microbial co-metabolite of choline, trimethylamine-*N*-oxide (TMAO), decreased. Lower urinary TMAO may reflect a lower intake of choline, consistent with reduced excretion of the endogenous choline metabolite, dimethylglycine (DMG). The urinary excretion of metabolites was altered post weight loss with increased excretion of lactate and the TCA cycle intermediates, citrate and succinate.

### Effects of the VLCD-induced weight loss on the fecal microbiome

The VLCD led to no overall changes in total microbiota diversity or bacterial count by PCR, but caused specific and potentially metabolically significant changes in a number of species. Given the possible relationships between the intestinal microbiome and metabolic states [11, 12], we examined the effects of the VLCD-induced weight loss upon the fecal microbiome. Mean total fecal bacterial DNA content was unaffected by the intervention (PreWL 1.23 ± 0.24 × 10^8^, post WL 1.40 ± 0.33 × 10^8^ 16S rRNA copies/mg feces, p = 0.64). The overall alpha diversity of the fecal microbiome, calculated as observed sequence variants; Faith’s phylogenetic diversity; and Shannon Index were also unchanged (paired t-test *p* > 0.05).

**Fig. 2:**
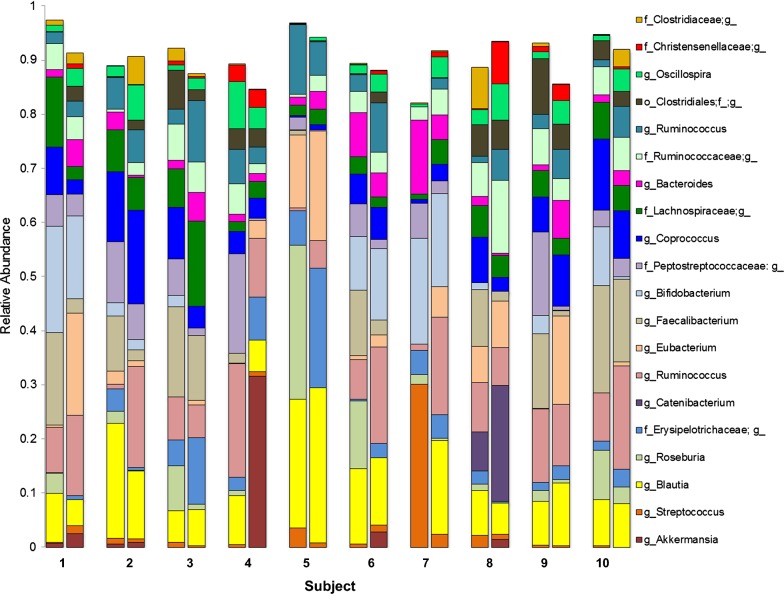
VLCD-induced weight loss modulates the fecal microbiome. Fecal samples were collected pre- and post-weight loss, 16S rRNA gene abundance determined via the MiSeq platform, and analyzed using QIIME 2. Microbial composition detected before and after the VLCD for the 20 most abundant taxa. The color key indicates specific bacterial taxa. All taxonomic analyses were performed at genus level, but some of the sequences detected were classified to groups with ambiguous genus-level names. Hence, *Clostridiaceae* does not actually indicate all members of the family *Clostridiaceae*, but rather all sequences classified as *Clostridiaceae*;g__ (i.e., family *Clostridiaceae* but unknown genus)

The 20 most abundant taxa in the fecal microbiota analysis comprise 91.3% of the PreWL sequences and 89.9% of the PostWL sequences. Plotting the 20 most abundant fecal taxa revealed marked heterogeneity between subjects, but substantial similarity between pre- and post-WL samples within each subject (Fig. 2). Several patterns were discernible. The inter-personal variation is greater than the variation due to treatment. Wilcoxon testing showed that the relative abundance of the genus *Roseburia* fell with the VLCD-induced weight loss (PreWL 0.070 ± 0.027, post WL 0.005 ± 0.003, p = 0.001), whereas *Christensenellaceae*;g__ (unknown genus) rose (PreWL 0.005 ± 0.003, post WL 0.017 ± 0.007, p = 0.032). Although taxa *Akkermansia* and *Streptococcaceae*;g__ as a proportion of total microbiota were quite low, a bloom of *Akkermansia* was found in the PostWL specimen from subject 4 and of *Streptococcaceae*;g__ in the PostWL specimen in Subject 6. The ratio of Bacteroidetes to Firmicutes did not change significantly (PreWL 0.06 ± 0.07 versus PostWL 0.07 ± 0.0.05, p = NS) despite the sustained weight loss.

**Fig. 3:**
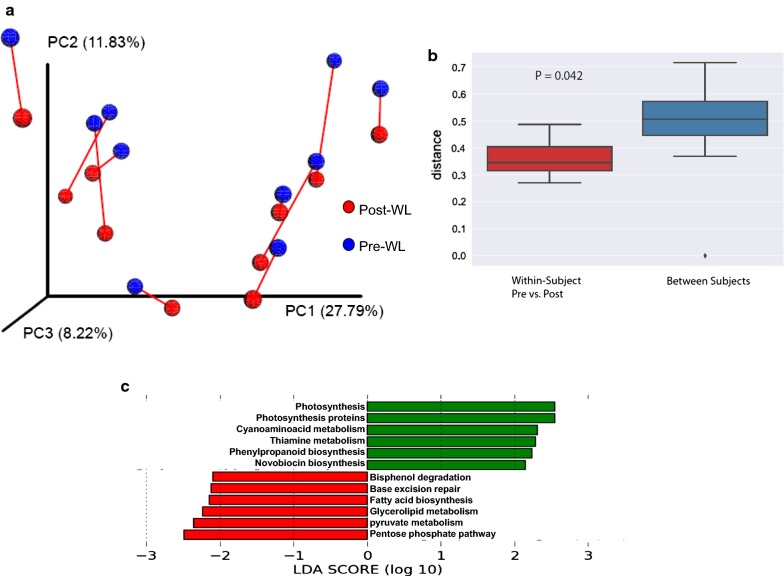
Diet induced weight loss shows conservation of the core gut microbiome. **a** Principal coordinate analysis (PCoA) of microbiome variation in pre-(blue circles) and post-(red circles) weight loss samples based on unweighted UniFrac analysis. The red lines connect the pre- and post-weight loss samples for each subject. For each subject, the positions along PC1 and PC3 did not change pre- and post-weight loss, whereas PC2 increased. **b** Average UniFrac Distance within subjects (red) versus between subjects (blue). **c** Imputed characterization of VLCD-induced gut functional changes induced by weight loss, as determined by Phylogenetic Investigation of Communities by Reconstruction of Unobserved States (PICRUSt) analysis. Colors indicate imputed bacterial pathways that decrease (green) or increase (red) with weight loss

The effect of the intervention on the community structure of the fecal microbiota was analyzed by unweighted and weighted UniFrac analysis, which assesses the degree of phylogenetic similarity among fecal samples, and was visualized by PCoA. In unweighted UniFrac analysis, three principal coordinates (PCs) account for 48% of the variability (Fig. 3a). The greatest differences between the individual study subjects were captured by PC1. The effects of the VLCD weight loss regimen were chiefly captured along PC2. Across all subjects, there was a consistent shift along PC2 when comparing pre- to post-WL samples (paired t-test, p < 0.001), indicating parallel shifts in community composition. This was also observed with weighted UniFrac analysis (paired t-test, p < 0.0002) (data not shown). Mean unweighted UniFrac distances were significantly lower within subjects versus between subjects (Fig. 3b), as represented by the PCoA results. There was no consistent shift in beta-diversity between the PreWL- and PostWL groups (PERMANOVA p > 0.05).

PICRUSt-imputed microbial functional analysis (Fig. 3c) showed relative enrichment of bacterial genes involved in fatty acid biosynthesis and glycerolipid metabolism, as well as pyruvate and pentose phosphate metabolism. These findings suggest that in response to the reductions in dietary carbohydrate and fat in the VLCD, there was selection for intestinal bacterial pathways related to the synthesis and metabolism of nutrients that were depleted.

### Associations between changes in the plasma metabolome and the fecal microbiome

**Fig. 4:**
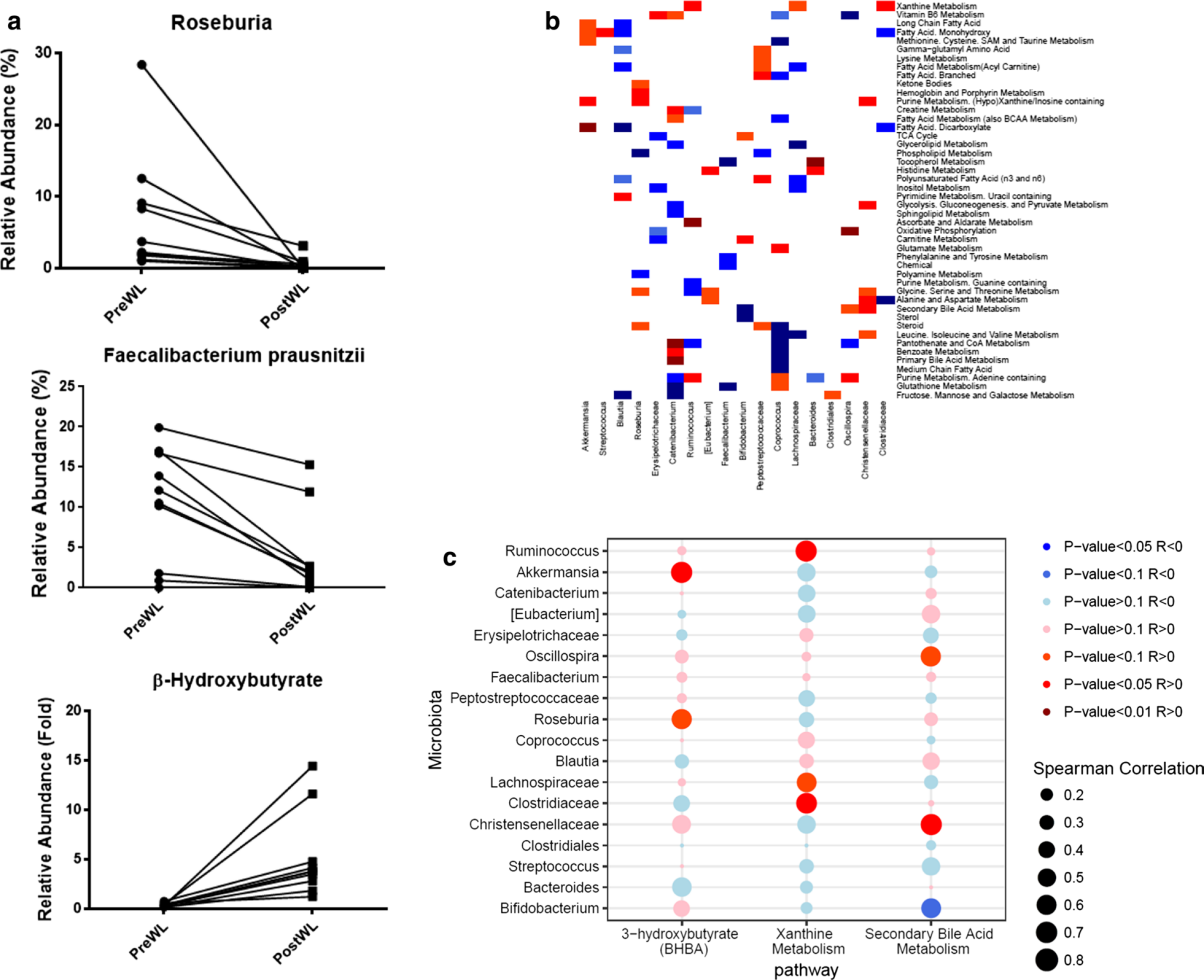
Coordinated effects of VLCD-induced weight loss on the plasma metabolome and the fecal microbiome. **a** VLCD-induced weight loss is associated with changes in both selected fecal bacterial taxa and levels of plasma β-hydroxybutyrate. **b** Spearman correlation heat map for bacterial taxa (rows) versus plasma metabolites (columns) changing with weight loss at *p*-value < 0.1. Red denotes positive correlation (p < 0.1), dark red denotes strongly positive correlation (p < 0.05), blue denotes negative correlation (p < 0.1) while dark blue denotes strongly negative correlation (p < 0.05). **c** Spearman correlation bubble plot of total fecal microbiota versus selected plasma metabolite pathways

The intestinal microbiota, modulated by the gut environment, produces bioactive metabolites such as short-chain fatty acids and bile acids. The most abundant bacterial taxa that decreased with the VLCD intervention were *Faecalibacterium prausnitzii* (PreWL 0.103 ± 0.023, PostWL 0.038 ± 0.017, p = 0.009) and genus *Roseburia* (PreWL 0.070 ± 0.027, PostWL 0.005 ± 0.003, p = 0.001) (Fig. 4a), which were negatively correlated with plasma BHB, a ketone body that is also a short chain fatty acid byproduct.

To further explore associations between gut microbiota and circulating metabolites following VLCD, we correlated changes in the plasma metabolome and the fecal microbiome (Fig. 4b). Metabolomic pathways that were significantly altered as reported [9], were also correlated with significant changes in the fecal microbiota (Additional file 2). There were strong positive correlations between *Catenibacterium* and primary bile acid metabolism (R = 0.82, p = 0.004), and *Christensenellaceae*;g__ with secondary bile acid metabolism (R = 0.68, p = 0.032). Purine metabolism pathways were strongly positively correlated with changes in *Roseburia* (R = 0.75, p = 0.018), *Ruminococcus* (R = 0.71, p = 0.027), and *Oscillospira* (R = 0.71, p = 0.027). We also observed negative correlations of *Erysipelotrichaceae*;g__ in metabolite pathways involved with oxidative phosphorylation (R = − 0.81, p = 0.008), TCA cycle (R = − 0.67, p = 0.039) and inositol metabolism (R = − 0.70, p = 0.031) in response to the VLCD weight loss. Xanthine metabolism was positively correlated with *Ruminococcus* (R = 0.67, p = 0.039).

We next sought to analyze the relative impact of the fecal microbiome in determining changes in circulating metabolites and metabolite pathways (Fig. 4c). We selected the metabolite BHB, which showed the largest increase in plasma PostWL, and two pathways involving the metabolism of biologically active intermediaries (xanthine and secondary bile acids) for correlation analysis with differentially changed bacterial taxa. The microbiota has greater impact on the metabolite 3BHB and the xanthine metabolism pathway than on the secondary bile acid metabolism pathway. The strong increase in plasma 3BHB was predominantly associated with positive changes in *Roseburia* and *Akkermansia*.

### Effects of the VLCD-induced weight loss on bile acids

**Fig. 5:**
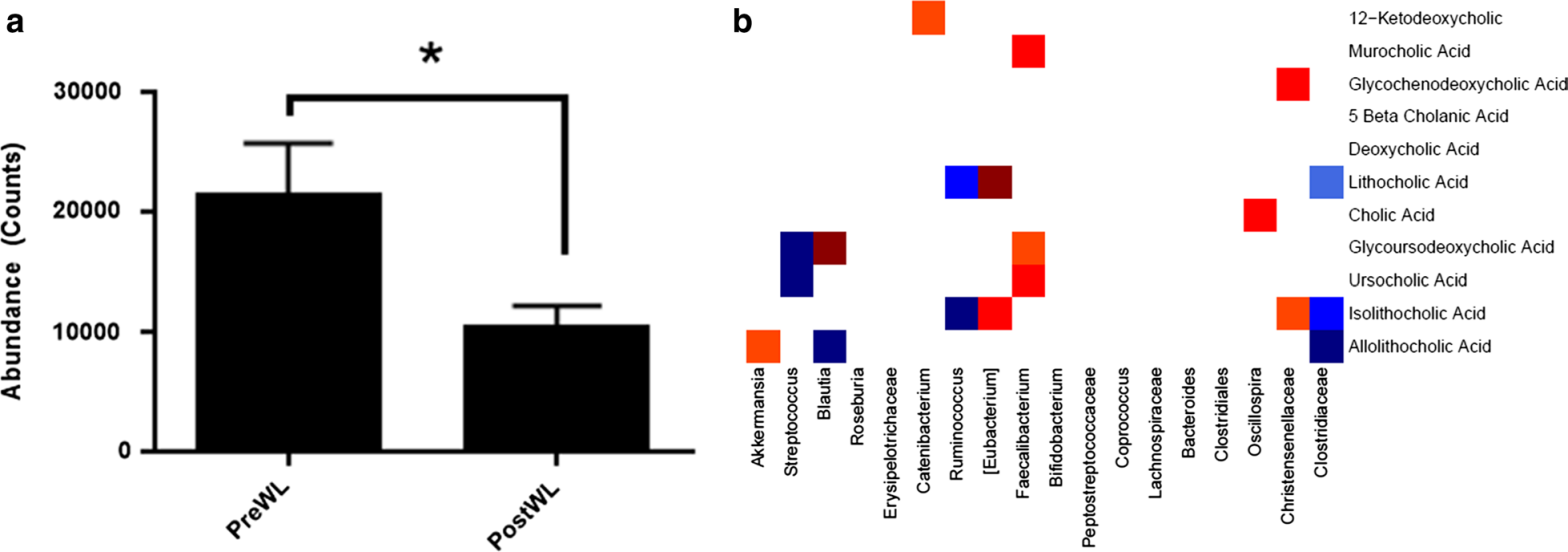
Coordinated effects of VLCD on fecal bile acids and microbiota. **a** Changes in total bile acids in feces following VLCD-induced weight loss (mean ± SE, *p < 0.05). **b** Spearman correlation heat map for bacterial taxa (rows) versus fecal bile acids (columns) changing with weight loss, at *p*-value < 0.1. Red denotes positive correlation (p < 0.1), dark red denotes strongly positive correlation (p < 0.05), blue denotes negative correlation (p < 0.1) while dark blue denotes strongly negative correlation (p < 0.05)

Since obesity is accompanied by fecal bile acid composition changes [15], we evaluated the effects of the VLCD-induced weight loss on the content and composition of fecal bile acids. Total fecal bile acid levels decreased by 26% after the intervention (Fig. 5a). Most (> 95%) fecal bile acids were unconjugated in both pre- and post-study samples. Approximately 50% deoxycholic acid (DCA) and ~ 40% of lithocholic acid (LCA) species were observed at baseline, and the mean percent of both DCA and LCA fell markedly after weight loss. They were partially replaced by increased primary (cholic and chenodeoxycholic) bile acids, suggesting reduction in bacterial deconjugation and subsequent oxidative enzymatic modification (Table 1). However, the distribution of these bile acids in the feces of individual subjects varied markedly, and changes were not significant.

**Table 1:** Composition of fecal bile acids detected by UPLC–MS (abundance)

	PreWL	Percent	PostWL	Percent	Paired T test
Conjugated BA					
Taurocholic	76		90		
Taurochenodeoxycholic	21		61		
Glycochenodeoxycholic	74		344		
Glycoursocholic	13		127		
Glycolithocholic	32		8		
Total conjugated BA	216	1.2	620	4.8	0.08
Unconjugated BA					
Lithocholic	5573	30.6	2372	18.4	0.11
Isolithocholic	1399	7.7	7.2	5.5	0.14
Muricholic + 3-β-muricholic	114	0.6	66	0.5	0.47
Cholic	1049	5.8	3796	29.5	0.47
Deoxycholic	9306	51.1	4721	36.7	0.07
Ursocholic	565	3.1	550	4.3	0.97
Total unconjugated BA	18,222	98.8	12,847	95.5	0.07
Total bile acids	18,625	100	13,786	100	0.02

Abundance is presented a counts from UPLC–MS analysis in fecal specimens

*PreWL* preweight loss, *PostWL* post weight loss, *BA* bile acid

Following weight loss, plasma bile acids showed an 8.7-fold increase in glycolithocholic acid and > threefold increase in taurolithocholic acids, with a twofold reduction in the cholic acid (Additional file 1: Table S1). Plasma FGF19 levels were variable and did not significantly change following VLCD-induced weight loss (PreWL 162 ± 39 pg/mL, PostWL 187 ± 53 pg/mL, p = NS).

### Associations between changes in fecal bile acids and the microbiome

The microbiota and bile acids in the gut lumen have reciprocal compositional effects [37]. We therefore examined bacterial taxa and individual bile acids in feces. We observed a strong negative correlation between *Clostridiaceae*;g__ (unknown genus) and LCA (Fig. 5b; R = − 0.82, p = 0.004) (Table 1), but strong positive correlations between *Eubacterium* and LCA (R = 0.82, p = 0.007), isolithocholic acid (R = 0.78, p = 0.012). There was also a strong positive correlation between *Oscillospira* with cholic acid (R = 0.660, p = 0.044). Isolithocholic correlated negatively with *Clostridiaceae*;g__ (R = − 0.706, p = 0.023) and *Ruminococcus* (R = − 0.78, p = 0.012). Taxa *Faecalibacterium* showed strong positive correlations with ursodeoxycholic acid (R = 0.72, p = 0.024) and murocholic acid (R = 0.67, p = 0.03). The observed bile acid changes point to possible bacterial-metabolite interactions with potential endocrine effects.

### Associations between changes in fecal bile acids and plasma metabolites

**Fig. 6:**
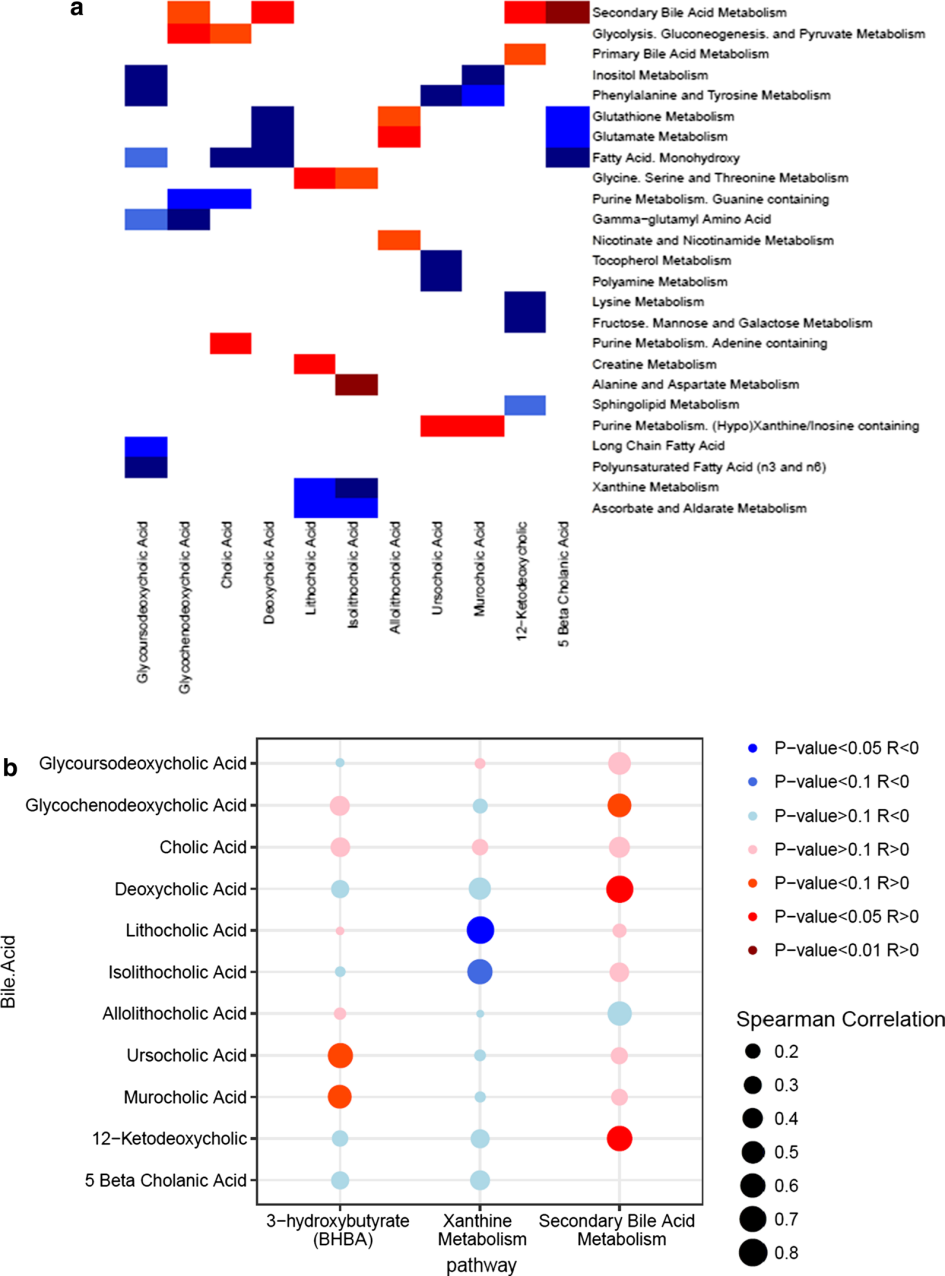
Coordinated effects of VLCD-induced weight loss on fecal bile acids and the plasma metabolome. **a** Spearman correlation heat map for plasma metabolites (rows) versus stool bile acids (columns) changing with weight loss at *p*-value < 0.1. Red denotes positive correlation (p < 0.1), dark red denotes strongly positive correlation (p < 0.05), blue denotes negative correlation (p < 0.1) while dark blue denotes strongly negative correlation (p < 0.05). **b** Spearman correlation bubble plot of total fecal bile acids versus selected plasma metabolite pathways

Bile acids in the gastrointestinal tract can alter circulating metabolites affecting glucose and lipid metabolism and homeostasis. These metabolic effects may occur directly through the action of circulating bile acids or indirectly through activation of receptors in the small or large intestine. We analyzed correlations between changes in circulating plasma metabolic pathways and changes in fecal bile acids (Fig. 6a). LCA correlated most strongly with the metabolic pathway for alanine and aspartate metabolism (R = 0.82, p = 0.007). The LCA species, lithocholic and isolithocholic acids, correlated positively with the metabolism of amino acids glycine, serine, and threonine (R = 0.672, p = 0.039; R = 0.6, p = 0.073, respectively). There was a positive correlation between purine metabolic pathways and ursocholic (R = 0.770, p = 0.014) and cholic (R = 0.721, p = 0.024) acids. Conversely, several bile acids correlated negatively with fatty acid monohydroxylate metabolism including glycoursodeoxycholic acid (R = − 0.85, p = 0.006), 5-β cholanic acid (R = − 0.610, p = 0.066) and DCA (R = − 0.610, p = 0.066). Strong negative correlations were found between xanthine pathway metabolites and LCA (R = − 0.781, p = 0.012). Sphingolipid metabolites also showed a strong negative correlation with 12-ketodeoxycholic acid (R =  − 0.794, p = 0.010). Secondary bile acid metabolism pathways, encompassing most circulating bile acids, correlated positively with fecal bile acids 5-β cholanic acid (R = 0.806, p = 0.008), 12-ketodeoxycholic acid (R = 0.660, p = 0.044) and, to a lesser extent, glycochenodeoxycholic acid (R = 0.563, p = 0.096). The concentration of fecal short chain fatty acids measured by GC–MS were quite low before the start of the VLCD and remained low thereafter (acetate 5 ± 3 mM, butyrate 1 ± 4 mM, propionate 6 ± 15 mM). A bubble plot illustrating the relative correlation of fecal bile acids with these selected plasma metabolites (Fig. 6b) shows a relationship with secondary bile acid metabolism. Similarly little effects on BHB and xanthine metabolism were observed.

### Association between fecal bile acids and the adipose tissue transcriptome

**Fig. 7:**
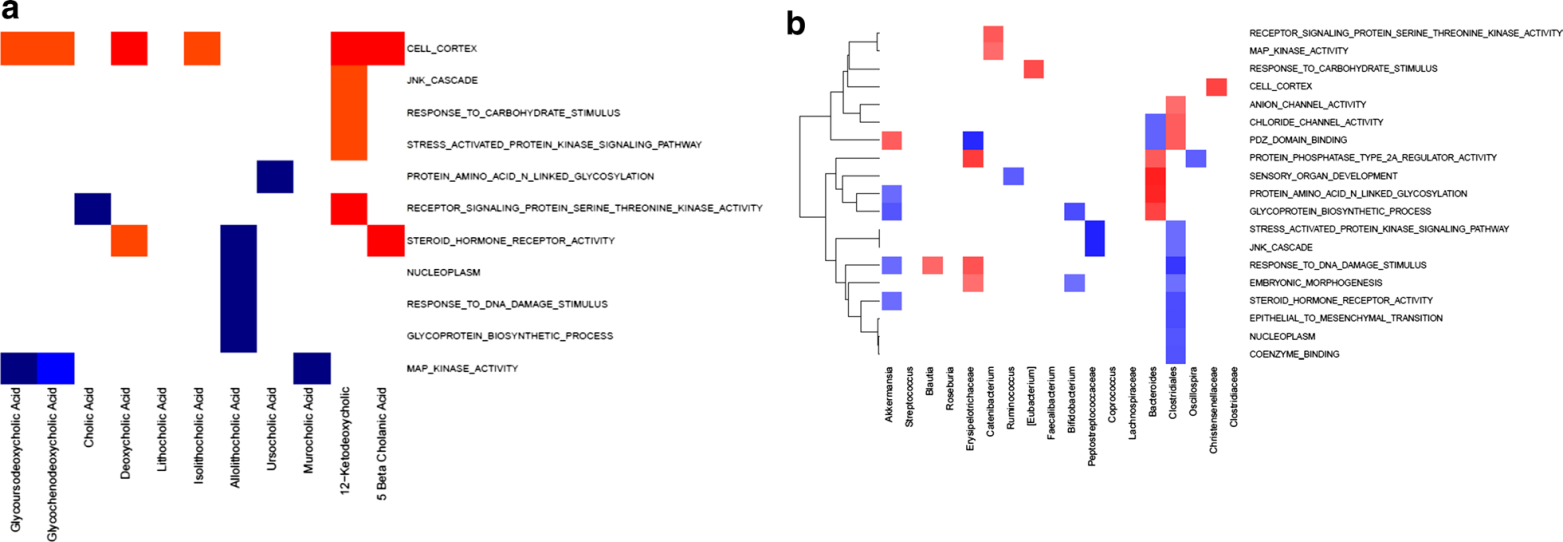
Coordinated effects of VLCD-induced weight loss on the fecal compartment and the adipose tissue transcriptome. **a** Spearman correlation heat map for adipose tissue transcriptome pathways (rows) versus fecal bile acids (columns) changing with weight loss, at *p*-value < 0.1. Red denotes positive correlation (p < 0.1), dark red denotes strongly positive correlation (p < 0.05), blue denotes negative correlation (p < 0.1) while dark blue denotes strongly negative correlation (p < 0.05). **b** Spearman correlation heat map for fecal microbiota taxa (rows) versus adipose tissue transcriptome pathways (columns) changing with weight loss, at p-value < 0.1. Red denotes positive correlation (p < 0.1), dark red denotes strongly positive correlation (p < 0.05), blue denotes negative correlation (p < 0.1) while dark blue denotes strongly negative correlation (p < 0.05)

We previously reported several metabolic changes in the white adipose tissue (WAT) transcriptome following VLCD-induced weight loss [9]. Correlation analysis with the newly presented fecal bile acid data shows WAT mitogen-activated protein (MAP) kinase gene expression negatively correlated with fecal glycochenodeoxycholic acid (R = − 0.66, p = 0.044; Fig. 7a). Other relationships of functional significance were the positive correlations between 12-deoxyketocholic acid and pathways for receptor signaling protein serine threonine kinase (R = 0.76, p = 0.016) and for cell cortex (R = − 0.70, p = 0.031). We also observed strongly positive correlations between 3 fecal bile acids (DCA and its metabolites, 5-β-cholanic and 12-ketodeoxycholic acids) and adipose tissue cell cortex genes (p < 0.05). Fecal microbiota also showed striking positive correlations with several gene expression pathways in adipose tissue including protein amino acid n-linked glycosylation (p < 0.005) (Fig. 7b). *Peptostreptococcaceae*;g__ was correlated negatively with the JNK cascade and stress activated protein kinase signaling pathways in adipose tissue (each p < 0.005). These results allow for further exploration of connections between intestinal contents and distant organs such as adipose tissue and should contribute to a better understanding of metabolic diseases.

## Discussion

The present study aimed to determine the effects of rapid weight loss induced by a VLCD on concomitant changes in fecal microbiota and bile acid content and composition. We also sought to characterize their relationship with the plasma metabolome and adipose tissue transcriptome. Urinary metabolomic profiles confirmed that starvation ketosis and protein sparing occurred during this intervention as well as a modulation of the functional status of the microbiome [38]. We show that the core microbiota was preserved during VLCD-induced weight loss, but we also found changes in several groups of bacterial taxa with functional implications. In parallel, fecal bile acid content fell, with a trend to increased fecal conjugated bile acids and reduced deoxycholic acid. These changes correlated with changes in plasma metabolites and adipose tissue gene expression and imply that changes in the fecal microbiome participate in many of the benefits of weight reduction.

Gut bacteria play an important role in metabolic health and metabolic homeostasis [39]. The fecal microbiota in baseline specimens differed markedly between the study subjects. However, despite consuming an identical VLCD diet in both composition and quantity, the fecal microbiota among the study subjects did not become more similar. Their individual core microbiome was preserved, and microbial richness was not reduced. A low microbial richness has been described in obese subjects with complications [10] and is reversible with a low calorie diet [40]. We did not observe these findings, possibly due to the population studied which was comprised of obese postmenopausal women with relatively few complications of their disease. Our data are consistent with observations that an abrupt but modest early change in fecal microbiota composition occurs shortly after a switch in diet, which reverted back toward the baseline microbiota following longer-term feeding of the new diet [41]. It is possible that assessment of the fecal microbiota earlier in VLCD-induced weight loss might have shown different results.

Due to our study design, changes attributable to weight loss *versus* diet change cannot be distinguished. Reports on the effects of diet-induced weight loss on the fecal microbiome have been mixed: some studies have shown conservation [42], while others report shifts [43] in the core microbiome. In contrast, bariatric surgery-induced weight loss is accompanied by dramatic shifts in microbiome composition, which might reflect both changes in gastrointestinal anatomy as well as nutrient effects [17].

We found several specific changes in the fecal microbiome that could have importantly affected systemic metabolism. In our study, *Christensenellaceae*;g__ (unknown genus) was relatively enriched between study start and study end. *Christensenella* is associated with healthy BMI, reduces diet-induced weight gain in mice, and is receiving lots of attention as a potentially heritable member of the microbiome [44]. It is surprising that in our study this bacterial genus belonging to *Christensenellaceae* negatively associated with weight loss and may be a potentially important finding. Any role of this genus in weight management may be more complex than we appreciate from cross-sectional studies. Prior studies have examined weight associations with this genus in obesity [45, 46] but do not associate it with weight loss, especially in very obese patients. On the other hand, *Faecalibacterium prausnitzii* decreased in our study, but has been proposed as a microbial discriminant between gut disorders as ulcerative colitis and Crohn’s disease [47]. There was no consistent increase in *Akkermansia*, as had been described [41]. Our subjects were very obese without complications, yet showed a reduced *Bacteroides*:*Firmicutes* ratio, which was unchanged by the VLCD. Our observation contrasts with prior studies [11, 42], indicating that this reduced ratio and its responsiveness to caloric restriction are not universal phenomena.

PICRUSt-imputed metagenomic functional analysis showed changes that may signify a response of the fecal microbiome toward increased carbohydrate and lipid metabolism following the intervention. The VLCD is severely restricted in carbohydrates and lipids, which should decrease the amounts of these nutrients entering the colon. Our analysis suggests that the microbiota may compensate for the lack of substrates in the diet by increasing their production of these carbohydrates and lipids following the VLCD [48]. Recent studies show that the quality of food rather than caloric content is the key for maintaining rich/healthy fecal microbiome [14, 49]. Although others observed changes in fecal SCFA content following VLCD-induced weight loss, as has recently been reported [42], these changes presumably reflect the lack of fermentable fiber in the colon.

Altered increased bile acid synthesis and transport has been reported to occur in obesity [15], and in our study total fecal bile acids decreased significantly following the VLCD. This decrease could result from lower hepatic bile acid synthesis and/or bile acid fractional turnover [50]. Fat-free diets reduce bile acid synthesis rates in humans [50] and have been shown to decrease fractional bile acid turnover in the rat [51] so that VLCD-induced decrease in fecal bile acids likely was due to both factors. In addition, VLCD feeding has been associated with reduced gall bladder emptying [52], which can contribute to increased gallstone formation that has been observed with this diet [53]. As expected, most fecal bile acids in our study were unconjugated with predominant LCA species. LCA is formed from chenodeoxycholic acid in the terminal ileum and colon mainly by the highly abundant genus *Clostridium* [54]. Although LCA has also been described to increase with caloric restriction [55] this did not occur after the VLCD in our study.

Bile acids interact with several receptors in gut epithelial cells [56], which induce metabolic effects in the intestine and the liver [19, 57]. Primary bile acids function as natural ligands for the gut nuclear receptor farnesoid X receptor (FXR) and secondary bile acids are ligands for the G protein coupled intestinal plasma membrane receptor TGR5. Activation of these receptors by bile acids can alter lipid [58], glucose [59], and energy homeostasis [60]. FXR inhibition improves obesity related metabolic dysfunction including insulin resistance [61]. In the mouse, microbial reduction of tauro-β-muricholic acid regulates ileal FGF15 concentrations and inhibits hepatic bile acid synthesis [62], illustrating the profound effect of the gut microbiota on bile acid metabolism. In addition, bile acid responsive receptors as well as bile acids [52] are found outside the enterohepatic circulation, including the kidney and heart [63]. The major elevation of fasting plasma levels of glycolithocholic and taurolithocholic sulfate that occurred after the intervention in our study may have interacted with such receptors to alter cellular metabolic functions. Gut bile acids activate FXR, which induces expression of FGF19 in humans [64]. Plasma FGF19 levels varied widely between subjects in our study, as previously reported [65] and in line with the between-subject variation in core fecal microbiome. Overall, our data suggest that VLCD-induced changes in circulating bile acid levels contribute to the beneficial effects of weight loss despite large variation between subjects.

Our weight loss regimen greatly impacted the metabolic homeostasis of our subjects as judged by its effects on fasting plasma metabolites [9]. Urinary metabolite abundance, which mirror starvation ketosis and decreased muscle protein catabolism, showed expected changes suggestive of muscle sparing. These metabolite changes could result from direct effects on the liver and internal organs or might reflect changes in gut microbiota or bile acids. Our data showed prominent increases in urinary as well as plasma content of 4CS, a microbial breakdown product of tyrosine and phenylalanine, to 4-hydroxyphenylacetate. This suggests greater availability of the precursors tyrosine and phenylalanine for fecal microbial metabolism. The presence of this microbial-derived metabolite implies additional crosstalk between amino acid absorption from the gut microbiota and its metabolism in the liver. Additionally, we observed decreased plasma abundance and urinary excretion of TMAO, which likely reflects the decrease in dietary red meat intake.

The branched chain amino acids (BCAAs) leucine, isoleucine, and valine are early biomarkers of insulin resistance with possible pathophysiologic roles in glucose intolerance and muscle insulin resistance [66, 67]. Changes in the levels of these BCAAs could be due to alterations in microbiota. We observed decreases in plasma levels of the BCAAs, tyrosine, and phenylalanine levels along with increases in urine isoleucine, 3-methyl-2-oxovalerate, and 3-hydroxyvalerate excretion. Plasma BCAA metabolism showed a trend toward a negative correlation with the fecal taxa *Lachnospiraceae*;g__ and *Coprococcus*. A recent European report also associated *Prevotella copri* with increased circulating BCAA levels in insulin resistance [18]. *Bacteroides thetaiotaomicron* has been linked to changes in BCAAs accompanying improved insulin resistance with weight loss [22]. However, these taxa were not in our top 20 differentially changed microbiota taxa. The TCA cycle metabolite citrate increased in plasma, whereas both urinary citrate and succinate levels decreased following the VLCD. *Erysipelotrichaceae;g__* correlated with TCA cycle and oxidative phosphorylation metabolite pathways.

Since microbiota and bile acids mutually interact in the gut, it is not surprising that we found highly significant positive correlations between plasma bile acid metabolism and fecal microbiota including *Catenibacterium* and *Christensenellaceae;g__*. *Catenibacterium* was enriched in subjects with improved CVD risk in the Bogalusa Heart Study [68]. *Christensenellaceae* has been associated with lower BMI [44]. *Roseburia* and *Ruminococcus* strongly correlated with plasma purine pathway metabolism. At the same time, *Ruminococcus* as well as *Christensenellaceae;g__* showed a positive correlation with xanthine metabolism, a pathway through which adenosine, adenine, and inositol are shunted into uric acid formation. Despite these microbial alterations, no difference was observed in plasma uric acid post VLCD-induced weight loss.

Plasma levels of BHB increased markedly after the intervention. This likely reflects systemic lipid metabolism, but could in part also result from less catabolism by *Faecalibacterium prausnitzii* and *Roseburia.* These organisms were negatively correlated with plasma BHB concentrations. Since conjugated bile acids activate FXR receptors expressed in adipose tissue [69], we further explored correlations between fecal bile acids (and fecal microbiota) and changes in WAT gene expression pathways. Serine threonine kinase, with its receptor, initiates the catabolism of ATP and participates in several metabolic pathways including MAP kinase signaling [70]. Therefore, the positive correlation between fecal 12-ketocholic acid and serine threonine receptor signaling suggests a functional effect. MAP kinase pathway gene expression also correlated with several fecal bile acids.

## Conclusion

**Fig. 8:**
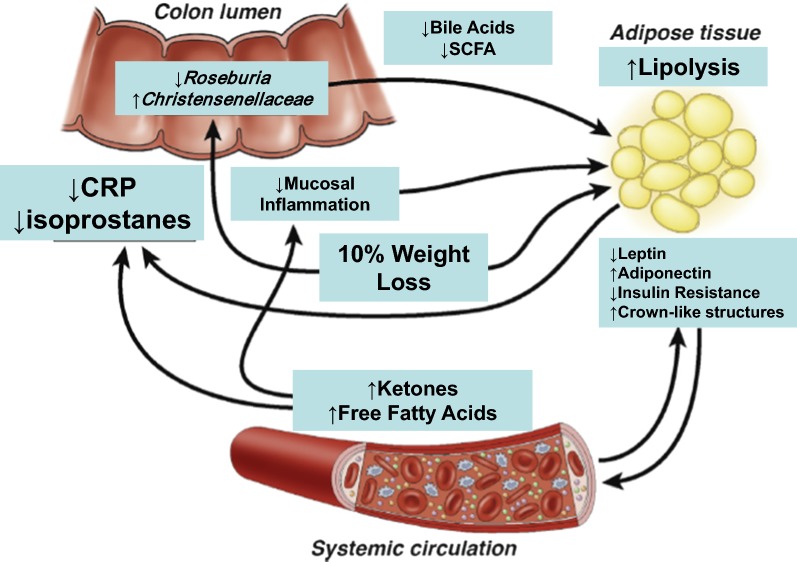
Potential mechanisms of fecal microbiota, fecal bile acid, plasma metabolome and adipose tissue transcriptome interactions in diet-induced weight loss (Adapted with permission from Aleman et al. Gastroenterology 2014)

VLCD-induced weight loss was used to obtain novel data on the changes in weight, microbiome, plasma and urinary metabolites, and adipose gene expression in humans. Though analysis of our clinical study was correlative, such information is essential to define the causes of many of the beneficial effects of weight loss. The strengths of the present study were that the environment and exercise for our subjects were carefully maintained and monitored, the diet was identical, and the degree of weight loss very similar. One weakness was that we studied only 10 subjects, so the associations need confirmation in model organisms or larger clinical studies. Pairwise correlations used in this study are limited in establishing causality for the observed associations. Another limitation of this study is the lack of fecal metabolite measurements. Due to feasibility considerations we did not include a weight neutral cohort to compare, but we previously reported on the stability of fecal microbiota following celecoxib treatment in a weight neutral study [34]. In summary, VLCD-induced weight loss modulated the fecal microbiome in functionally important ways to compensate for the lack of nutrients, with concomitant decreases in total fecal bile acids and most unconjugated species detected. Moreover, we show that some changes induced by the microbiome are likely to alter systemic metabolism, and have identified others that occur but do not correlate with either adipose or metabolite changes as outlined in Fig. 8. We posit the metabolic changes occurring in the host with caloric restriction and 10% weight loss, namely ketogenesis and free fatty acid production, have a profound systemic effects directly through metabolic hormonal signals and indirectly by inducing microbiota-related changes that signal through their own metabolites. Using a very controlled diet in a metabolic ward, we show diet specific changes in the fecal microbiome and fecal bile acid profile. By providing an intervention model that associates related compartments such as the fecal microbiota and fecal bile acids with distant compartments such as the plasma metabolome and subcutaneous adipose tissue, we propose a novel approach to the study of the biology of human weight loss.

## Additional files

**Additional file 1: Table S1.** Composition of plasma bile acids detected by LC–MS. **Table S2.** Urinary metabolite ratios following VLCD-induced weight loss. **Figure S1.** Urine metabolomic orthogonal projection to latent structures-discriminant analysis (OPLS-DA) identifying the metabolic variation associated with weight loss.

**Additional file 2.** Pairwise correlation tables. **Table S1.** Plasma metabolomic-fecal microbiota correlations. **Table S2.** Fecal bile acid-fecal microbiota correlations. **Table S3.** Plasma metabolomic-fecal bile acid correlations. **Table S4.** Fecal bile acid-adipose transcriptomic pathway correlations. **Table S5.** Fecal microbiota-adipose transcriptomic pathway correlations.

## Abbreviations

4CS: 4-cresyl sulfate
BCAA: branched chain amino acid
BHB: β-hydroxybutyrate
CLS: crown-like structures
DCA: deoxycholic acid
DMG: dimethylglycine
FXR: farnesoid X receptor
GC–MS: gas chromatography–mass spectrometry
hsCRP: high-sensitivity C-reactive protein
LCA: lithocholic acid
LC–MS: liquid chromatography–mass spectrometry
MAP: mitogen-activated protein
OPLS-DA: orthogonal projection to latent structures-discriminant analysis
PAG: phenylacetylglutamine
PC: principal coordinates
PCoA: principal coordinate analysis
PLS-DA: partial least squares-discriminant analysis
PostWL: post weight loss
PreWL: preweight loss
TMAO: trimethylamine-*N*-oxide
UPLC: ultra performance liquid-chromatography
UPLC–MS: ultra performance liquid chromatography–mass spectrometry
VLCD: very low calorie diet (VLCD)
WAT: white adipose tissue
