# Supplementary material for: Fecal microbiota and bile acid interactions with systemic and adipose tissue metabolism in diet-induced weight loss of obese postmenopausal women
Source: J Transl Med. 2018 Sep 3;16:244. doi: 10.1186/s12967-018-1619-z (PMC6122649; doi:10.1186/s12967-018-1619-z)
Supplement: Supplementary file 1 — Additional file 1: Table S1. Composition of plasma bile acids detected by LC–MS. Table S2. Urinary metabolite ratios following VLCD-induced weight loss. Figure S1. Urine metabolomic orthogonal projection to latent structures-discriminant analysis (OPLS-DA) identifying the metabolic variation associated with weight loss. [file 12967_2018_1619_MOESM1_ESM.docx]

Additional file 1: Table S1: Composition of Plasma Bile Acids detected by LC-MS

| Sub Pathway | Biochemical Name | Mean Pairs Ratio PostWL/PreWL | p-value* |
| --- | --- | --- | --- |
| Primary Bile Acid Metabolism | cholate | **0.45** | 0.0069 |
|  | glycocholate | 0.98 | 0.2694 |
|  | taurocholate | 1.24 | 0.6578 |
|  | glycochenodeoxycholate | 0.90 | 0.1562 |
|  | taurochenodeoxycholate | 1.13 | 0.5756 |
| Secondary Bile Acid Metabolism | glycodeoxycholate | 3.70 | 0.4889 |
|  | taurodeoxycholate | 3.26 | 0.1237 |
|  | glycolithocholate sulfate* | **8.76** | 0.0263 |
|  | taurolithocholate 3-sulfate | **3.11** | 0.0014 |
|  | glycocholenate sulfate* | 1.08 | 0.8788 |
|  | taurocholenate sulfate* | **2.36** | 0.0618 |

*p-value calculated as paired t-tests accounting for multiple comparisons.

Additional file 1: Table 2: Urinary Metabolite Ratios following VLCD-Induced Weight Loss

| Urinary Metabolite | Mean Pairs Ratio PostWL/PreWL | PostWL vs PreWL (OPLS correlation coefficient) |
| --- | --- | --- |
| 1, 3-methyl-2-oxovalerate | 1.54 | 0.77 |
| 2, pantothenate | 1.60 | 0.89 |
| 3, 2-aminobutyrate | 1.53 | 0.85 |
| 4, isoleucine | 1.30 | 0.83 |
| 5, unknown 1 | 1.64 | 0.85 |
| 6, 3-hydroxyisovalerate | 1.30 | 0.59 |
| 7, lactate | 1.21 | 0.87 |
| 8, unknown 2 | 1.77 | 0.88 |
| 9, alanine | 0.81 | -0.71 |
| 10, acetone | 2.89 | 0.76 |
| 11, acetoacetate | 2.50 | 0.49 |
| 12, succinate | 1.77 | 0.88 |
| 13, citrate | 1.73 | 0.66 |
| 14, unknown 3 | 0.52 | -0.76 |
| 15, methylguanidine | 0.74 | -0.78 |
| 16, dimethylglycine | 0.75 | -0.82 |
| 17, creatine | 0.61 | -0.67 |
| 18, malonate | 0.70 | -0.60 |
| 19, trimethylamine-*N*-oxide | 0.71 | -0.59 |
| 20, unknown 4 | 0.37 | -0.90 |
| 21, unknown 5 | 0.71 | -0.75 |
| 22, 3-methylhistidine | 0.20 | -0.85 |
| 23, guanidinoacetic acid | 0.64 | -0.88 |
| 24, tartrate | 1.76 | 0.76 |
| 25, 4-cresyl sulfate | 1.75 | 0.62 |
| 26, phenylacetylglutamine | 1.42 | 0.66 |
| 27, salicylurate | 1.79 | 0.78 |
| 28, *N*-methyl-2-pyridone-5-carboxamide | 1.45 | 0.56 |
| 29, *N*-methylnicotinamide | 1.57 | 0.68 |


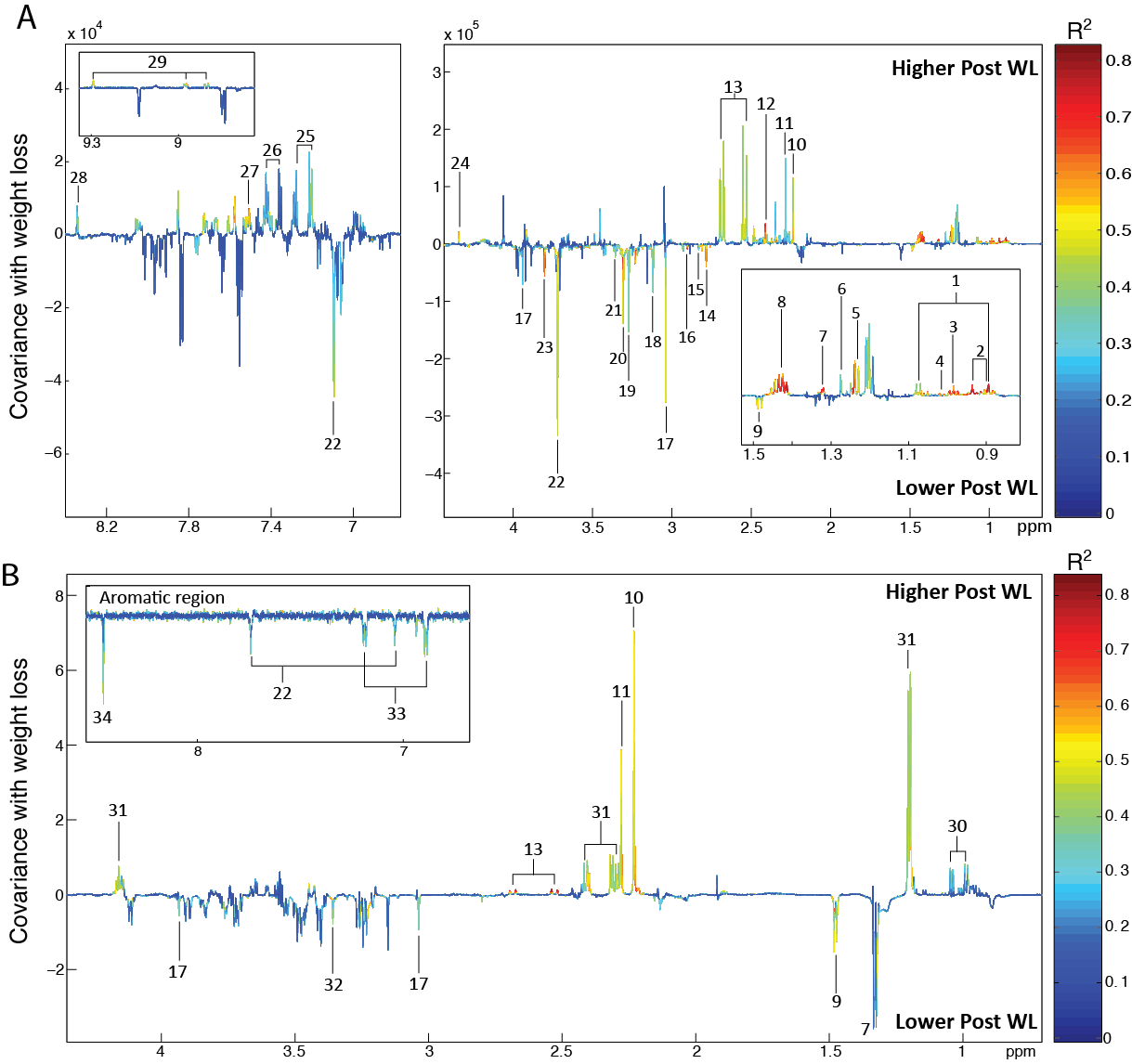


Additional file 1: Figure S1: Urine metabolomic orthogonal projection to latent structures-discriminant analysis (OPLSDA) identifying the metabolic variation associated with weight loss. Coefficient plots extracted from the OPLSDA models built on the urinary metabolome (Q^2^Y = 0.56). The spectra identify 29 compounds with upward (indicating increase with weight loss) or downward deflection (decrease with weight loss). A. NMR Spectra from PPM 8.4-6.8. B. NMR Spectra from PPM 4.5-0.5. The corresponding compounds are: Key: 1, 3-methyl-2-oxovalerate; 2, pantothenate; 3, 2-aminobutyrate; 4, isoleucine; 5, unknown 1; 6, 3-hydroxyisovalerate; 7, lactate; 8, unknown 2; 9, alanine; 10, acetone; 11, acetoacetate; 12, succinate; 13, citrate; 14, unknown 3; 15, methylguanidine; 16, dimethylglycine; 17, creatine; 18, malonate; 19, trimethylamine-*N*-oxide; 20, unknown 4; 21, taurine; 22, 3-methylhistidine; 23, guanidinoacetic acid; 24, tartrate; 25, 4-cresyl sulfate; 26, phenylacetylglutamine; 27, salicylurate; 28, *N*-methyl-2-pyridone-5-carboxamide; 29, *N*-methylnicotinamide
